# Properties of orb weaving spider glycoprotein glue change during *Argiope trifasciata* web construction

**DOI:** 10.1038/s41598-019-56707-1

**Published:** 2019-12-30

**Authors:** Brent D. Opell, Sarah D. Stellwagen

**Affiliations:** 1 Department of Biological Sciences, Virginia Tech, Blacksburg, VA USA; 2 Department of Biological Sciences, University of Maryland, Baltimore County, Baltimore, MD USA

**Keywords:** Biophysics, Ecology, Zoology, Materials science

## Abstract

An orb web’s prey capture thread relies on its glue droplets to retain insects until a spider can subdue them. Each droplet’s viscoelastic glycoprotein adhesive core extends to dissipate the forces of prey struggle as it transfers force to stiffer, support line flagelliform fibers. In large orb webs, switchback capture thread turns are placed at the bottom of the web before a continuous capture spiral progresses from the web’s periphery to its interior. To determine if the properties of capture thread droplets change during web spinning, we characterized droplet and glycoprotein volumes and material properties from the bottom, top, middle, and inner regions of webs. Both droplet and glycoprotein volume decreased during web construction, but there was a progressive increase in the glycoprotein’s Young’s modulus and toughness. Increases in the percentage of droplet aqueous material indicated that these increases in material properties are not due to reduced glycoprotein viscosity resulting from lower droplet hygroscopicity. Instead, they may result from changes in aqueous layer compounds that condition the glycoprotein. A 6-fold difference in glycoprotein toughness and a 70-fold difference in Young’s modulus across a web documents the phenotypic plasticity of this natural adhesive and its potential to inspire new materials.

## Introduction

**Figure 1:**
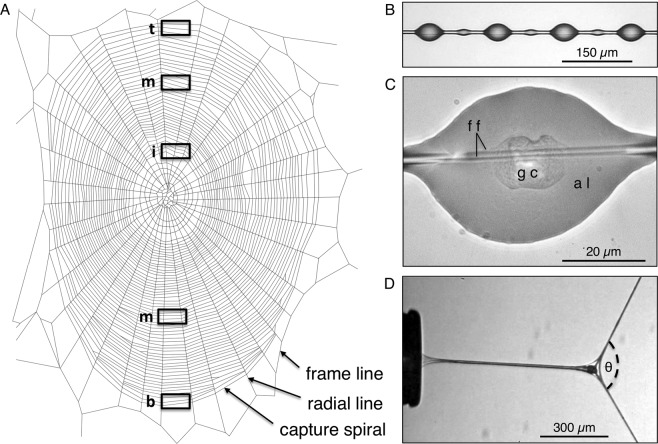
Orb web features. (**A**) Tracing of an *Argiope trifasciata* web identifying thread components and showing in rectangles the five positions from which capture thread samples were taken (t = top, m = middle, i = inner, b = bottom). (**B**) A short strand of viscous prey capture thread showing glue droplets. (**C**) A flattened thread droplet, showing flagelliform axial fibers (ff), glycoprotein core (gc), and aqueous layer (al). (**D**) An extending thread droplet being pulled from the tip of a probe, identifying the angular deflection (ϴ) of the support line.

Engineers and chemists increasingly turn to biological systems for inspiration as they develop new applications and products^1–3^. Spider threads, particularly major ampullate threads, owing to their great toughness, have received much attention in this regard^4,5^. These proteinaceous threads are the product of abdominal glands whose silk is encoded by MaSp1 and MaSp2 genes of the spidroin family^6–9^. When an *Argiope trifasciata* (Forskål, 1775) spins its web in the high humidity of the pre-dawn hours, these major ampullate threads are the first to be deposited, forming the web’s frame and radial elements (Fig. 1A) that will serve as a scaffold for the spirally arrayed prey capture thread (Fig. 1A,B)^10–13^. The frame and radial lines of the completed web absorb and dissipate the force of a prey strike^14–16^, while its prey capture threads retain an insect until a spider can locate, run to, and begin wrapping it with silk^17^.

Unlike a web’s dry major ampullate threads, its viscous capture thread is a composite thread, comprised of the products of two kinds of spinning glands. Each of the spider’s paired posterior lateral spinnerets possess a flagelliform spigot that is flanked by two aggregate gland spigots. As a proteinaceous flagelliform fiber is drawn from the spigot, it is coated with aggregate gland solution^18^. The coated fibers from each spinneret merge to form an aggregate cylinder, which contains amorphous proteins as well as organic and inorganic low molecular mass compounds (LMMCs)^19,20^. Plateau-Rayleigh instability^21,22^ then causes this cylinder to quickly form into a regular series of droplets (Fig. 1B). A glycoprotein core forms with each droplet^23,24^ and the rest of the aggregate material remains as an aqueous layer. This layer covers the glycoprotein core and the flagelliform fibers, both those that extend through the glycoprotein and those in inter-droplet regions (Fig. 1C). The LMMCs make the aqueous layer hygroscopic, ensuring that both glycoprotein and flagelliform fibers remain hydrated and plastic (Fig. 1D). Other proteins that are not easily visible under standard light microscopic examination remain in the aqueous layer^25^. In this study, we refer to the material at the center of a droplet as its glycoprotein (Fig. 1C).

In contrast to other biological adhesives, such as those used by mussel and barnacles to adhere to surfaces that are secreted as low viscosity solutions and subsequently stiffen^26–30^, orb weaver glycoprotein remains viscoelastic^31–33^. Combined with flagelliform fiber extensibility, this creates a compliant adhesive delivery system. As a series of adhering droplets extend (Fig. 1D), their adhesive forces are transferred to the thread’s bowing support line and summed in suspension bridge fashion^34,35^. Consequently, glycoprotein adhesion and extensibility have coevolved along with the aqueous layer’s LMMCs, to ensure that, at a spider’s foraging humidity, the glycoprotein is viscous enough to establish adhesive contact, but cohesive enough to transfer force during extension^36^.

Both the AgSp1 and AgSp2 spidroin genes that code a droplet’s glycoprotein^37–40^ and the glycoprotein’s biomimetic potential are receiving attention^41,42^. However, due to the small size of a droplet’s glycoprotein core and the fact that its performance is conditioned by LMMCs in the surrounding aqueous layer, only recently has a technique been developed to characterize the glycoprotein’s material properties in its native condition^43^.

A droplet’s consolidated glycoprotein appears to account for most of its adhesive performance^31^. However, LMMCs in the droplet’s aqueous layer make important contributions to its adhesive function beyond conferring droplet hygroscopicity that cause droplet size and performance to change over the course of a day as they track environmental humidity^20,44^. LMMCs also solvate and maintain glycoprotein structure, increasing its surface interaction^25,45^ and they remove interfacial water from a droplet’s contact footprint, enhancing adhesion^46^.

An adult female *A. trifasciata* deposits an average 23.2 m of capture thread when constructing a web^47^. This capture thread is the web’s most costly component, accounting for as much as 80% of the web’s dry mass^48^, with the greatest portion being comprised of aggregate silk gland material (Fig. 1C). Consequently, stores of this material and the cost of its synthesis may be a limiting factor in the web’s production^49–51^. As a result, a spider’s nutritional state can affect the properties of its capture thread and its ability to retain prey^52–54^. Capture thread properties may also change during web construction in ways that affect prey capture, either as a result of material depletion, or allocation to mitigate this. The objective of this study is to test this hypothesis by characterizing the volumes and material properties of the glycoprotein glue cores from different regions of *A. trifasciata* webs.

Large orb webs, like those spun by *A. trifasciata*, exhibit a top-bottom asymmetry, with the area below the web’s hub being larger than the area above^55,56^. This results in part from the spider initiating capture thread placement with a series of switchback turns at the bottom of the web before transitioning to continuous circular deposition of threads (Fig. 1A). Consequently, the sequence of capture thread deposition is from the bottom of the web to the periphery of the web, to the inner spiral turns. As *A. trifasciata* preferentially feeds on large insects that can more easily struggle from a web^57^, the time required to reach different web regions can affect prey capture success. This species rests facing downward in the center of its web and, like other large orb weavers, can run downward faster than upward, thereby minimizing the difference in the time required to reach top and bottom web sectors in order to begin wrapping prey to prevent their escape from the web^55^*. Argiope trifasciata* places its webs in weedy vegetation where lower web regions are more likely to intercept orthopterans^57^. This results in these spiders capturing more of these large prey than do species whose webs are place higher in vegetation^58^.

These observations suggest that it could be adaptive for *A. trifasciata* to allocate aggregate gland material differently across web regions. However, this would require a spider to assess its aggregate gland supply (either before constructing a web or before initiating a capture spiral), assess environmental humidity (which affects the size and distribution of forming droplets^21^), and dispense aggregate gland material accordingly, perhaps also altering capture spiral spacing. Alternatively, the use of aggregate material may be unrestricted because aggregate gland size and web construction behavior have coevolved to ensure that changes in material properties either occur in regions of the web where they impact prey retention the least, or where any negative effects are mitigated by changes in other web features.

We hypothesize that the allocation of aggregate gland material during web construction is unrestricted and that diminishing gland reserves impart the properties to capture thread glue droplets from different web regions. The alternate hypothesis is that a spider either has an excess of aggregate gland material or that it allocates this material differentially across a web. We test this hypothesis by comparing the properties of capture threads from the bottom switchback region, top, middle, and inner regions of *A. trifasciata* webs to determine if they change during web construction (Fig. 1A). We cannot directly determine if intra-web differences that we may find are attributable to depletion or to allocation of aggregate gland resources. However, if these resources are not limited or are allocated across a web, then the variance in droplet and glycoprotein properties in different web regions should be similar. If depletion of aggregate material underlies these differences then disparity in the nutritional and reproductive status of spiders included in our study should result in increased variance in glue droplet properties as capture thread deposition progresses, because some spiders will begin to deplete their aggregate gland reserves sooner than others.

## Results

**Figure 2:**
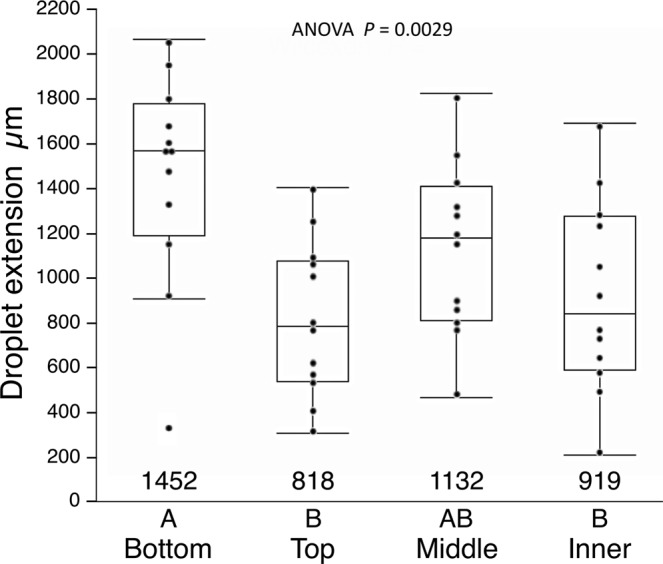
Lengths of extended glue droplets from four web positions at pull-off. Letters above the web positions are Tukey-Kramer rankings of mean values, which appear below the box plots.

**Figure 3:**
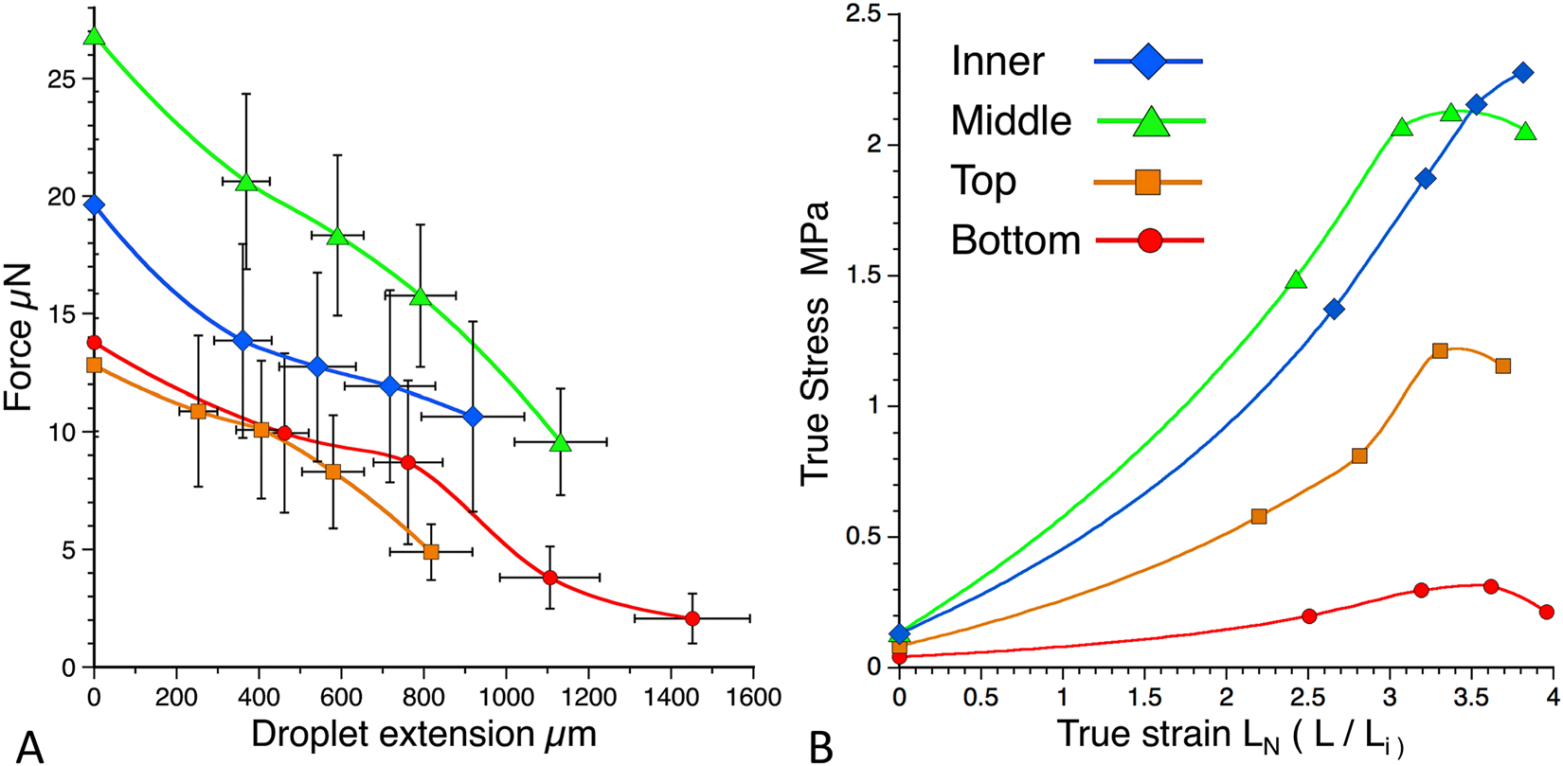
Force – extension (**A**) and True stress–True strain (**B**) curves of glycoproteins from four web positions. Error bars in force–extension curves are ± 1 standard error.

The length of droplet extension decreases during spiral deposition (Fig. 2), although the force on droplets during extension is greater in the latter than in the earlier stages of spiral deposition (Fig. 3A). In this species, force decreases as extension progresses (rather than increases, as is normally the case) because sufficient force must be exerted to cause the flattened glycoprotein to form a short cylinder before extension can be observed and measured. As the glycoprotein extends, its cross-sectional area decreases and progressively less force is required to extend the droplet. When the force–extension curve is translated into a true stress–true strain curve, force on the filament is divided by the glycoprotein filament’s cross-sectional area to yield true stress, which causes true stress to increase during extension until the glycoprotein’s elastic region is exceeded (Fig. 3B). Because the glycoprotein is under stress before extension begins, true stress is greater than zero at a true strain of zero, with this difference increasing as the glycoprotein becomes stiffer during web construction.

**Figure 4:**
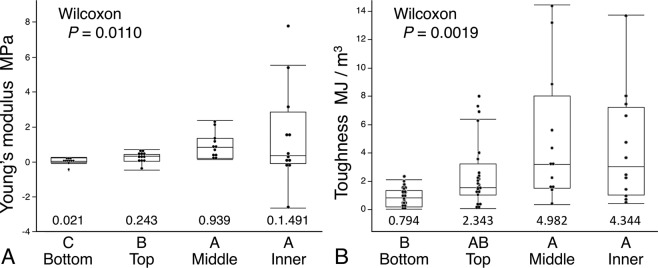
Young’s modulus (**A**) and Toughness (**B**) of glycoprotein from four web positions. Ranking letters above the web positions are from Wilcoxon each pair tests of mean values, which appear below the box plots.

**Figure 5:**
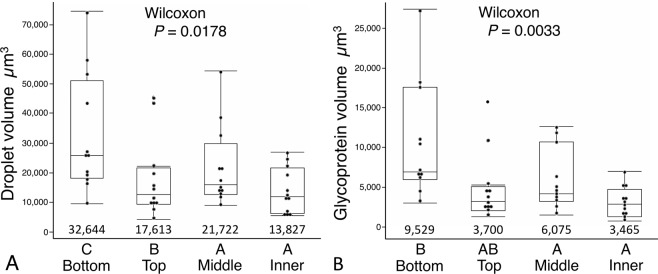
Volumes of droplets (**A**) and their glycoproteins (**B**) from four web positions. Ranking letters above the web positions are from Wilcoxon each pair tests of mean values, which appear below the box plots.

**Figure 6:**
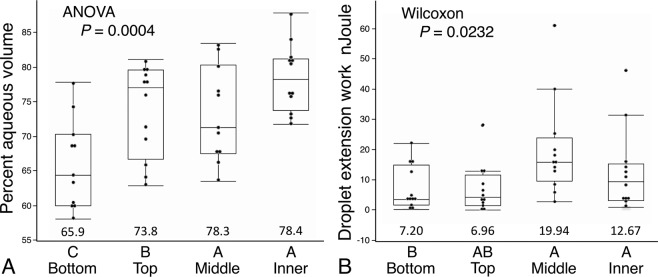
Percent aqueous volume (**A**) and work of extension (**B**) of droplets from four web positions. Ranking letters above the web positions are from a Tukey-Kramer test (Percent aqueous volume) and Wilcoxon each pair tests of mean values (Droplet extension work), which appear below the box plots.

Both the Young’s modulus and toughness of glycoprotein increase as web construction advances (Fig. 4A,B). Although this increase appears to be progressive, Wilcoxon each pair tests identified no differences between the values of middle and inner web regions. Both droplet volume and glycoprotein volume decrease during web construction, with the greatest drop being between the bottom and top web positions (Fig. 5A,B). The percent of droplet aqueous volume increases during web construction (Fig. 6A), indicating an increase in droplet hygroscopicity and water content, thus providing no evidence that increases in glycoprotein Young’s modulus and toughness during web construction resulted simply from a decrease in glycoprotein hydration. The decrease in a droplet’s glycoprotein volume during web construction (Fig. 5B) coupled with an increase in glycoprotein toughness (Fig. 4B), resulted in top and bottom web positions exhibiting lower work of droplet extension than middle and inner web positions (Fig. 6B).

Analysis of Means for Variances tests with Alpha of 0.05 showed that the standard deviation (SD) of bottom and top web position Young’s modulus (0.24 and 0.31 MPa, respectively) were less than the overall mean SD of 1.53 MPa, that middle position SD (0.80 MPa) did not differ from the mean, and that inner position SD (2.87 MPa) exceeded the mean. Bottom position toughness SD (0.64 MJ/m^3^) was less than the overall mean SD of 3.33, MJ/m^3^, top and inner poison toughness (2.34 and 4.01 MJ/m^3^, respectively) did not differ from the mean, and middle position toughness (4.75 MJ/m^3^) slightly exceeded the mean’s upper confidence level of 4.73 MJ/m^3^. Bottom position droplet volume SD (6359 µm^3^) was greater than the overall mean SD of 4138 µm^3^, whereas top, middle, and inner position SD (3076, 3618, and 2344 µm^3^, respectively) did not differ from mean SD. The SD of work of droplet extension of bottom, top, middle, and inner positions (7.33, 7.92, 16.10, and 13.49 nJ, respectively) did not differ from the overall mean SD of 11.81 nJ.

Supplementary Table 1 provides the means and standard errors of droplet values, Supplementary Table 2 the values used to construct true stress–true strain curves, and Supplementary Table 3 the material properties of glycoproteins from each web position.

**Figure 7:**
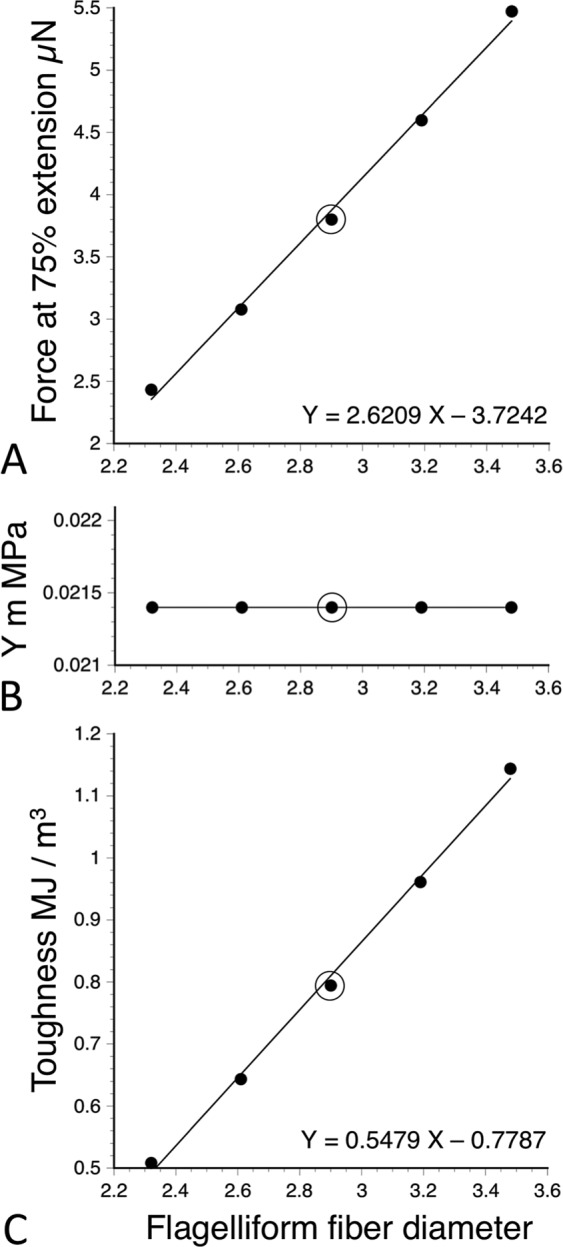
The effects of changing flagelliform fiber diameter from the 2.9 µm (circled) value that was used in intra-web comparisons on bottom web position glycoprotein filament force at 75% extension (**A**), glycoprotein Young’s modulus (Y m) (**B**), and glycoprotein toughness (**C**).

As described in *Methods*, we use the properties of a capture thread’s flagelliform fibers to gauge the force on an extending glycoprotein filament. Consequently, changes in flagelliform fiber diameter (FFD) during web construction could affect our results. We examined this issue by changing (FFD) in the computations that we used to determine glycoprotein Young’s modulus and toughness. When the FFD of bottom position threads was changed by 10% increments, force on the glycoprotein filament changed (Fig. 7A). However, because these changes shift the entire stress–strain curve relative to the Y stress axis, the slope of the curve and, thus, the Young’s modulus derived from it was not affected (Fig. 7B). Consequently, differences in FFD across a web would not affect the Young’s modulus values that we report. However, changing FFD did affect glycoprotein toughness (Fig. 7C). Changing the FFD of top, middle, and inner web positions to achieve glycoprotein toughness values equivalent to that of the bottom web position required FFDs of 1.6664, 1.1710, and 1.2390 µm, respectively. These values were 57%, 40%, and 43%, respectively, of the 2.9 µm FFD that we used to compute the glycoprotein toughness of all web positions. Differences in FFD of this magnitude are unlikely to occur across a single orb web, making it improbable that intra-web differences in FFD fully explain the differences in glycoprotein toughness that we observed.

## Discussion

As hypothesized, the properties of glue droplets change during web construction. Increases in the variance of glycoprotein Young’s modulus and Toughness during web construction (Fig. 4A,B) are consistent with the explanation that this results from depletion of aggregate gland material rather than a conservative allocation of this material. These changes might be viewed as a spider simply running low on resources and, therefore, non-adaptive. However, this spinning process does impart a pattern to the properties of the web’s glue droplets, and this pattern may be integral to the evolution of the web’s characteristics. Therefore, as we learn more about other web features, such as glue droplet distribution and spiral spacing, it may be possible to determine if web construction behavior has evolved to incorporate these changes in ways that complement or compensate for the intra-web differences in glue droplet properties that we observed.

The reduction in flagelliform fiber diameter (FFD) required to make glycoprotein toughness equal across a web was 13% greater than the difference between *A. trifasciata* and *A. aurantia* FFD^16^. Moreover, FFDs required to equalize glycoprotein toughness across an *A. trifasciata* web would require the diameters to be similar to those of much smaller orb weaving spiders. The mean body mass of seven orb weaving species studied with FFDs of 1.1–1.8 µm (values in the range of those necessary to equalize glycoprotein toughness across an *A. trifasciata* web) averaged 44 mg^16^. This contrasts with *A. trifasciata*, which, for Blacksburg, Virginia, USA area populations, had a mean mass of 474 mg^59^ and 581 mg (Opell, unpublished observations 2019). Additionally, when *Nephila clavipes* and *Argiope keyserlingi* were put on a low protein diet for five or ten days, the volumes of their droplets and their glycoprotein cores increased^52,54^, whereas flagelliform fiber diameter remained unchanged. In the latter study, *Nephila plumipes* fiber diameter increased slightly on the low protein diet. This indicates that flagelliform fiber properties are less prone to nutritional influence than are other capture thread properties and, therefore, are not likely to change during the course of a single web’s construction, particularly to the extent required to explain the differences in glycoprotein toughness that we observed.

Experimentally altering humidity during web construction is known to affect the size of forming droplets^21^. However, the *A. trifasciata* threads that we studied were produced under natural conditions during pre-dawn hours when humidity ranges from 95–100% RH^32,60^, making it very unlikely that humidity differed across the web during capture thread deposition. Instead, progressive changes in the composition of aggregate gland material appear to account for these differences. Of an orb weaver’s seven types of silk glands, the aggregate glands have, by far, the largest volume^61^. Changes in the processing of aggregate material as it passes down the duct from the gland to spinning spigot^62^ may also contribute to these differences. In the aqueous layers of spiders deprived of food, the molar percentages and absolute quantities of less readily synthesized compounds (e.g., choline, isethionate, N-acetyltaurine) decrease, while more readily synthesized compounds (e.g., GABamide and glycine) increase^53^. As these less readily synthesized compounds tend to be more hygroscopic^19,20^, a decrease in droplet hygroscopicity, and therefore volume, might be expected to accompany aggregate gland depletion. Although we did observe a decrease in droplet volume during web construction, aqueous layer volume percentage increased 21% from bottom to inner droplets (Fig. 6A). Thus, the hygroscopicity of a droplet’s aqueous layer appears to increase during web construction. Consequently, increases in glycoprotein Young’s modulus and toughness during web construction cannot be attributed simply to reduced glycoprotein viscosity resulting from a reduction in the aqueous layer’s water content. However, this leaves open the possibility that there was a decrease in the concentration of some LMMCs that are crucial for plasticizing glycoprotein, but contribute little to droplet hygroscopicity, while the concentration of other very hygroscopic LMMCs increased. Although the hygroscopicities of LMMCs have been characterized^20^, their specific contributions to glycoprotein conditioning is unknown.

Biological materials can exhibit considerable inter-specific differences in their properties. For example, the toughness of major ampullate threads of common North American orb weaving species range from 102–254 MJ/m^3^. However, that of Darwin’s bark spider from Madagascar can attain a toughness of 520 MJ/m^3^ ^16^. Consequently, in assessing potential applications of biological materials, it is important to understand the range of their properties and, when possible, factors that govern these differences. When measured at 55% RH and 23 °C, the toughness of three orb weavers differed greatly: *Argiope aurantia* - 0.28 MJ/m^3^, *Neoscona crucifera -* 1.71 MJ/m^3^, and *Verrucosa arenata -* 29.12 MJ/m^3^^43^. Within an *A. trifasciata* web, glycoprotein toughness showed a 6.27-fold difference, ranging from 0.7944 MJ/m^3^ at the bottom of the web to 4.9816 MJ/m^3^ in the middle of the web. Young’s modulus exhibited a 69.6-fold difference, ranging from 0.0214 MPa in bottom droplets to 1.4905 MPa in inner droplets.

Unlike the adhesives used by barnacles and mussels that harden after being secreted^26–28,30^, orb weaver glycoprotein glue relies on sustained viscoelasticity to function as a compliant adhesive^31,54,60,63^. Moreover, capture thread performance is optimized and glycoprotein viscosity tuned to the humidity of a spider’s habitat during its foraging period^36^. Therefore, it is not surprising that the material properties of orb spider glycoprotein exhibit inter-specific differences^43,63^. However, it more surprising to find significant intra-web differences in glycoprotein properties. These differences, along with differences in glue droplet distribution, capture spiral spacing, and web tension have the potential to affect the prey retention performance of different web regions (Fig. 5B). As this biological adhesive inspires new products, these intra-web differences appear to provide an ideal context for understanding factors that modulate orb spider glycoprotein glue properties.

## Methods

### Collecting and preparing threads

Sectors of webs of 12 adult female *A. trifasciata* were collected near Blacksburg, Virginia. We did this by placing a 15 × 52 cm rectangular aluminum frame vertically across the center of a web to capture the top and bottom portions of the web and inner spirals on the sides of the web. Double-sided tape (Cat. # 9086K29550360, 3 M Co., Maplewood MN, USA.) applied to a frame’s 1 cm faces secured the web sector and maintained native thread tension. Frames were transported and kept in closed boxes to prevent threads from being contaminated by dust and pollen and were stored in the laboratory at 50% RH prior to testing. We collected web sectors between 05:30 h and 08:30 h from 15 September 2014 to 03 October 2014 and captured all images and videos described below by 16:00 h on the day of collection, ensuring that thread age did not impact our results.

As explained more fully in previous studies^33,35,43,64,65^, in the laboratory we used tweezers whose tips were covered in double-sided carbon tape (Cat #77816, Electron Microscope Sciences, Hatfield PA, USA) and blocked open to accommodate the spacing of U-shaped brass supports epoxied at 4.8 mm intervals to microscope slides with their free ends extending upward and covered with double-sided carbon tape (Fig. 3 in Opell *et al*., 2011). Capture spiral threads were collected from 5 places on each web (Fig. 1A): 1. The top most threads just beneath the upper frame thread, 2. The middle of the web’s top section, determined by dividing the total number of spirals in the top section in half, 3. The innermost threads located adjacent the web’s hub, 4. The middle of the web’s bottom section, and 5. The bottom-most spiral threads. Because it was not possible to identify differences in the spinning sequence between threads in the top middle and bottom middle web regions, the values of these two web positions were pooled in the analysis. Additionally, two-tailed, paired T-tests confirmed that the droplet properties of these two middle regions did not differ: Droplet volume (*P* = 0.93), Glycoprotein volume (*P* = 0.63), Young’s modulus (*P* = 0.35), Toughness (*P* = 0.98), and Work of Droplet Extension (*P* = 0.64).

### Determining glycoprotein volume

All droplet and glycoprotein measurements as well as all droplet extensions were conducted at 23 °C and 55% relative humidity. Using previously described techniques^33,35,43,64,65^, we determined the volumes of three droplets and their glycoprotein cores, using each individual’s mean values in statistical analyses. Mean values of the measurements used in these computations are reported in Supplementary Table 1. We measured the droplet length (DL; dimension parallel to the axial fiber) and width (DW) of suspended droplets used to compute droplet volume according to the following formula^66^.

1 $${\rm{DV}}=\frac{(2{\rm{\pi }}{\rm{\times }}D{W}^{2}{\rm{\times }}{\rm{DL}})}{15}$$

These three droplets were then flattened under a glass coverslip, making their glycoprotein core visible (Fig. 1C). We computed the volume of a droplet’s glycoprotein core by first dividing droplet volume by flattened droplet area to determine droplet thickness. We then multiplied droplet thickness by the surface area of the flattened glycoprotein core to determine glycoprotein volume. Each droplet’s glycoprotein volume to droplet volume ratio was determined and an individual’s mean value was then multiplied by the volumes of this individual’s extended droplets (see below) to infer the volumes of their glycoprotein cores. This was necessary because droplets can either be extended to measure their material properties, or flattened to measure their glycoprotein volumes, but not both.

As LMMCs solvate and stabilize glycoprotein^25,45^, changes in the amounts of these compounds during web spinning could account for any observed changes in glycoprotein Young’s modulus and toughness. Because LMMCs are also responsible for a droplet’s hygroscopicity^20^, one indication of such changes would be a change in droplet hygroscopicity during web construction as indicated by a change in the relative water content of a droplet. We evaluated this by characterizing the percent aqueous volume (PAV) of droplets from each web position using the following formula.

2 $$PAV=\frac{droplet\,volume-glycoprotein\,volume\,}{droplet\,volume}\times 100$$

### Extending droplets

Three droplets from each web position were individually extended to pull-off as described previously^33,35,43,64,65^ (Fig. 1D). This was done by first cleaning the 413 µm wide, polished steel tip of a probe with 100% ethanol, bringing it into contact with a droplet located at the center of a 4800 µm thread span, pressing the droplet against the probe until its support line was deflected by 500 μm, anchoring the probe, and then using a stepping motor to advance the X-axis of the Mitutoyo FS60 inspection microscope (Mitutoyo America Corp., Aurora IL, USA) stage on which the thread observation chamber rested, thus extending the droplet. We extended droplets at a velocity of 69.6 μm s^−1^ while 60-fps videos recorded their elongations and the deflections of their support lines (Fig. 1D).

Preparing droplets for extension entailed sliding adjacent droplets away from the droplet to be extended. To confirm that this did not withdraw material from the droplet that was extended, we used a two-way ANOVA to compare native droplet volume and the volume of droplets that had been prepared for extension from each of the four web regions. Although droplet volume differed among web position (*P* = 0.0007) neither native-extended droplet volumes nor the crossed effects of native-extended and web position droplet volumes differed (*P* = 0.5535 and 0.8238, respectively). Thus, there is no evidence that preparing droplets for extension altered their properties.

### Characterizing glycoprotein material properties

Using recently developed techniques^43^ and applied in a subsequent study^63^, we characterized the Young’s (elastic) modulus and toughness of *A. trifasciata* glycoprotein in droplets from each of the four web positions. We performed this analysis for Phase 1 extension^43^, the first portion of a droplet’s extension during which the extending glycoprotein filament is completely covered by aqueous material (Fig. 1D). This corresponds to droplet performance typically observed in the course of thread pull off^43^.

The angular deflection of the line supporting an extending droplet (Fig. 1D) was used in conjunction with the diameters (2.9 µm) and Young’s modulus (8 MPa) of the two flagelliform fibers within this line (values taken from the literature^16^): to compute the force on the glycoprotein filament within an extending droplet. As the details of this method are explained in the literature^43^, we only review the procedures here. When droplet extension was first initiated we measured the support line’s deflection angle and assigned a droplet length equal to the diameter of the droplet’s glycoprotein core when configured as a sphere. At each additional 25% extension interval we measured both filament length and support line angular deflection.

At each interval we computed true strain as the natural log of the droplet filament’s length divided by the diameter of its glycoprotein core when configured as a sphere. True stress on a filament was computed at the initiation of droplet extension and at the four subsequent 25% extension intervals. This was done by dividing the force on the filament (determined as described below) by glycoprotein cross sectional area (CSA). At the initiation of extension CSA was computed as that of a cylinder with a height equal to the diameter of a glycoprotein core when configured as a sphere. At the remaining five intervals, CSA was determined by dividing glycoprotein volume by filament length. An extended droplet was positioned at the center of a 4800 µm thread span. Therefore, we were able to use the Young’s modulus and diameters of the axial lines and the angular deflection of the support line to calculate the force that each side of the support line exerted on the droplet. This angle (Fig. 1D) was then used again to resolve these force vectors into force on the extending glycoprotein filament.

### Evaluating the effects of flagelliform fiber diameter on glycoprotein properties

We examined the effects of flagelliform fiber diameter (FFD) on glycoprotein Young’s modulus and toughness in two ways. First, we determined the effects of 10 and 20% reductions and 10 and 20% increases in the 2.90 µm FFD of bottom web sector force on glycoprotein filament and on glycoprotein Young’s modulus and toughness. We then successively altered FFD for all replicates of top, middle, and inner web positions until we arrived at an FFD for each position that yielded a grand mean toughness value for each web position equal to that of the bottom position value of 0.7944 MJ.m^3^. Differences between the FFD values necessary to achieve this match and the standard 2.90 µm used in our computations indicated the degree to which FFD would have to change during web construction to account for the intra-web differences in glycoprotein toughness that we report.

### Statistical analysis

Properties of the three replicate droplets used to characterize droplet and glycoprotein volumes and of the three extended droplets used to characterize glycoprotein material properties for each position of an individual’s web were averaged and these mean values used as this individual’s values for graphical and statistical comparisons. Thus, each web position had a sample size of 12 for statistical tests. We used JMP (SAS Institute, Cary NC, USA) to analyze data, considering comparisons with *P* ≤ 0.05 as significant. The normal distributions of values were assessed with a Shapiro-Wilk W-test, with values of *P* > 0.05 being considered normal. If one or more web position values were not normally distributed we used a Wilcoxon / Kruskal-Wallis Test to compare mean values and, when significant, then used Wilcoxon Each Pair test to rank mean position values. If position values were normally distributed, we used a one-way ANOVA to compare values and a Tukey-Kramer HSD test to rank means.

## Supplementary information

Supplementary Information.

## Data Availability

All data generated or analyzed during this study are available in the manuscript and its three Supplementary Tables.
