# Supplementary material for: Properties of orb weaving spider glycoprotein glue change during Argiope trifasciata web construction
Source: Sci Rep. 2019 Dec 30;9:20279. doi: 10.1038/s41598-019-56707-1 (PMC6937294; doi:10.1038/s41598-019-56707-1)
Supplement: Supplementary file 1 — Supplementary Information. [file 41598_2019_56707_MOESM1_ESM.docx]

**Properties of orb weaving spider glycoprotein glue change during *Argiope trifasciata* web construction**

Brent D. Opell and Sarah D. Stellwagen

**Scientific Reports**

**Supplementary Material**

Supplementary Table 1. Characteristics of *Argiope trifasciata* droplets at four web positions. Mean ± 1 standard error.

| Droplet Feature | Web position | | | |
| --- | --- | --- | --- | --- |
|  | Bottom | Top | Middle | Inner |
| Glyco. area & vol. No. | 12 ± | 12 | 12 | 12 |
| Droplet length μm | 54 ± 3 | 43 ± 3 | 47 ± 2 | 41 ± 2 |
| Droplet width μm | 36 ± 2 | 29 ± 2 | 32 ± 1 | 27 ± 2 |
| Droplet volume μm^3^ | 32,644 ± 5812 | 17,613 ± 3903 | 21,722 ± 3920 | 13,827 ± 2220 |
| Droplet area μm^2^ | 6,296 ± 763 | 4,238 ± 567 | 4,854 ± 343 | 3,897 ± 434 |
| Droplet thickness μm | 4.93 ± 0.40 | 3.76 ± 0.32 | 4.21 ± 0.25 | 3.55 ± 0.30 |
| Glycoprotein area μm^2^ | 1,981 ± 258 | 1,105 ± 184 | 1,272 ± 135 | 815 ± 102 |
| Glycoprotein vol. μm^3^ | 10,666 ± 2249 | 4,707 ± 1242 | 5,874 ± 901 | 3,075 ± 560 |
| Glyco. vol. / Drop. vol. | 0.34 ± 0.02 | 0.26 ± 0.02 | 0.26 ± 0.02 | 0.22 ± 0.01 |
| Extended droplets No. | 12 | 12 | 12 | 12 |
| Droplet length μm | 51 ± 3 | 40 ± 2 | 46 ± 3 | 41 ± 3 |
| Droplet width μm | 34 ± 2 | 27 ± 2 | 31 ± 2 | 28 ± 2 |
| Droplet volume μm^3^ | 28,030 ± 5066 | 13,882 ± 2788 | 21,098 ± 3993 | 16,036 ± 3178 |
| Inferred glyco vol μm^3^ | 9,529 ± 1,836 | 3,700 ± 888 | 6,075 ± 1,044 | 3,465 ± 677 |

Supplementary Table 2. *Argiope trifasciata* phase 1 axial line deflection, computed force on extended droplet, and droplet length from 25% to full extension at four web positions. Mean ± 1 standard error.

| *N* = 12 | Glycoprotein  Volume µm^3^ | | Axial Line Angle ° | Glycoprotein Filament Force µN | Droplet Length µm |
| --- | --- | --- | --- | --- | --- |
| **Pre**  **Extension** |  | |  |  |  |
| Bottom | | 9,529 ± 1,836 | 132 ± 3 | 13.78 ± 3.76 | 0 |
| Top | | 3,700 ± 888 | 133 ± 5 | 12.81 ± 3.03 | 0 |
| Middle | | 6,075 ± 1,044 | 115 ± 3 | 26.85 ± 4.44 | 0 |
| Inner | | 3,465 ± 677 | 126 ± 5 | 19.63 ± 4.82 | 0 |
| **25%**  **Extension** |  | |  |  |  |
| Bottom | 9,529 ± 1,836 | | 142 ± 5 | 9.94 ± 3.37 | 462 ± 59 |
| Top | 3,700 ± 888 | | 138 ± 6 | 10.86 ± 3.20 | 253 ± 46 |
| Middle | 6,075 ± 1,044 | | 122 ± 3 | 20.62 ± 3.72 | 369 ± 57 |
| Inner | 3,465 ± 677 | | 135 ± 5 | 13.85 ± 4.11 | 361 ± 70 |
| **50%**  **Extension** |  | |  |  |  |
| Bottom | 9,529 ± 1,836 | | 146 ± 5 | 8.70 ± 3.47 | 762 ± 84 |
| Top | 3,700 ± 888 | | 140 ± 6 | 10.08 ± 2.92 | 406 ± 61 |
| Middle | 6,075 ± 1,044 | | 125 ± 3 | 18.33 ± 3.41 | 591 ± 63 |
| Inner | 3,465 ± 677 | | 138 ± 6 | 12.74 ± 4.00 | 542 ± 93 |
| **75%**  **Extension** |  | |  |  |  |
| Bottom | 9,529 ± 1,836 | | 154 ±4 | 3.80 ± 1.32 | 1106 ± 121 |
| Top | 3,700 ± 888 | | 143 ± 6 | 8.30 ± 2.40 | 580 ± 75 |
| Middle | 6,075 ± 1,044 | | 130 ± 3 | 15.76 ± 3.03 | 792 ± 86 |
| Inner | 3,465 ± 677 | | 142 ± 6 | 11.92 ± 4.07 | 718 ± 110 |
| **100%**  **Extension** |  | |  |  |  |
| Bottom | 9,529 ± 1,836 | | 161 ± 4 | 2.06 ± 1.06 | 1452 ± 140 |
| Top | 3,700 ± 888 | | 150 ± 5 | 4.89 ± 1.18 | 818 ± 100 |
| Middle | 6,075 ± 1,044 | | 140 ± 4 | 9.56 ± 2.26 | 1132 ± 112 |
| Inner | 3,465 ± 677 | | 146 ± 7 | 10.64 ± 4.03 | 919 ± 125 |

Supplementary Table 3. *Argiope trifasciata* glycoprotein material properties. Mean ± 1 standard error.

| Web position  *N* = 12 | Young’s modulus  MPa | | Toughness  MJ/m^3^ | Work of Droplet Extension nJoule |
| --- | --- | --- | --- | --- |
| Bottom | | 0.0214 ± 0.0734 | 0.7944 ± 0.1857 | 7.1977 ± 2.1161 |
| Top | | 0.2429 ± 0.0898 | 2.3426 ± 0.6751 | 6.9557 ± 2.2852 |
| Middle | | 0.9388 ± 0.2400 | 4.9816 ± 1.3705 | 19.9416 ± 4.6490 |
| Inner | | 1.4905 ± 0.8291 | 4.3440 ± 1.1562 | 12.6731 ± 3.8931 |
